# Carotid Catheterization and Automated Blood Sampling Induce Systemic IL-6 Secretion and Local Tissue Damage and Inflammation in the Heart, Kidneys, Liver and Salivary Glands in NMRI Mice

**DOI:** 10.1371/journal.pone.0166353

**Published:** 2016-11-10

**Authors:** Anne Charlotte Teilmann, Björn Rozell, Otto Kalliokoski, Jann Hau, Klas S. P. Abelson

**Affiliations:** 1 Department of Experimental Medicine, Faculty of Health and Medical Sciences, University of Copenhagen, Blegdamsvej 3b, DK-2200, Copenhagen N, Denmark; 2 Department of Comparative Medicine, Karolinska Institutet, SE-171 77, Stockholm, Sweden; US Geological Survey, UNITED STATES

## Abstract

Automated blood sampling through a vascular catheter is a frequently utilized technique in laboratory mice. The potential immunological and physiological implications associated with this technique have, however, not been investigated in detail. The present study compared plasma levels of the cytokines IL-1β, IL-2, IL-6, IL-10, IL-17A, GM-CSF, IFN-γ and TNF-α in male NMRI mice that had been subjected to carotid artery catheterization and subsequent automated blood sampling with age-matched control mice. Body weight and histopathological changes in the surgical area, including the salivary glands, the heart, brain, spleen, liver, kidneys and lungs were compared. Catheterized mice had higher levels of IL-6 than did control mice, but other cytokine levels did not differ between the groups. No significant difference in body weight was found. The histology revealed inflammatory and regenerative (healing) changes at surgical sites of all catheterized mice, with mild inflammatory changes extending into the salivary glands. Several catheterized mice had multifocal degenerative to necrotic changes with inflammation in the heart, kidneys and livers, suggesting that thrombi had detached from the catheter tip and embolized to distant sites. Thus, catheterization and subsequent automated blood sampling may have physiological impact. Possible confounding effects of visceral damage should be assessed and considered, when using catheterized mouse models.

## Introduction

Serial blood sampling through a vascular catheter is used for pharmacokinetics (PK) and other studies that require multiple small blood volumes from mice. A novel computer assisted system for serial automated blood sampling (ABS) minimizes handling stress and potential difficulties associated with blood collection from mice [1–4]. However, few studies have evaluated the physiological effects of long-term catheterization and ABS in mice, and these have focused on stress parameters [5–7].

Even with careful aseptic surgical technique, fibrin forms at the catheter tip, promoting the formation of microthrombi [8,9] that may occlude the catheter, or be released to the circulation, with possible complications such as local vessel inflammation, sepsis or major organ damage [10–12].

Fonseca *et al*. and found kidney infection and inflammation in catheterized rats seven days after surgery [12]. In the ABS system, regular infusion of small aliquots of heparinized saline is employed to maintain catheter patency [13,14]. While this measure reduces or prevents development of large thrombi, we hypothesized that it increases the detachment of microthrombi that embolize to distant sites.

Tissue damage, arising from catheterization, initiates an inflammatory response with secretion of cytokines and acute phase proteins (APPs) to the circulation, promoting tissue inflammation and repair [15,16]. The catheter may mechanically injure the vessel endothelium, additionally contributing to the secretion of cytokines and APPs [17]. The elevation may be sustained further by stressors, such as the continuous connection to the ABS system and associated single housing [13,18,19]. The catheterized mouse undergoing ABS may be affected both systemically, by inflammatory mediators and stress, and in multiple tissues by damage arising from microthrombi.

Even if clinical effects are not evident, such sequela may affect animal welfare and confound experimental results. In the light of the increasing use of ABS in mice, e.g. in pharmacological and toxicological studies, it is important to understand the pathophysiologic effects that may influence study outcomes. The present study compared the inflammatory responses in catheterized mice, that had been subjected to ABS, with non-catheterized control mice by quantifying pro-inflammatory (IL-1β, TNF-α, IL-6) and immune regulatory (IL-10, IFN-γ, GM-CSF, IL-2, IL-17A) cytokines. Additional assessments included body weight measurements and histological evaluation of the surgical sites, salivary glands, heart, brain, spleen, liver, kidneys, adrenals and lungs. The hypothesis was that catheterized mice would express elevated concentrations of plasma cytokines, weigh less and demonstrate pathologic changes of highly vascularized organs, such as the heart, lung, liver and kidneys, in contrast to control mice.

## Materials and Methods

This study was conducted in a fully AAALAC accredited facility and approved by The Animal Experiments Council under the Danish Ministry of Environment and Food (license number: 2012/561-169).

The facility followed the FELASA guidelines [20] for routine health monitoring of mice and rats. The animals had tested positive for *Helicobacter sp*. but none of the other pathogens on the FELASA list.

### Animals and housing

Fifteen male BomTac:NMRI mice (Taconic, Ry, Denmark), eight control mice and seven catheterized mice, were submitted for pathology at 9–11 weeks of age following a preceding study on stress associated effects in relation to catheterization and ABS [7]. The number of mice although limited by the preceding study, was estimated to be adequate by means of a sample size calculation based on data from previous studies with α and β levels set at 0.05 and 0.80, respectively; an estimated mean difference in the concentrations of cytokines exceeding 30% was considered biologically relevant.

The mice were single-housed in either Macrolon type II cages (Techniplast, Buguggiate, Italy) (control mice) or cages associated with an ABS system (Dilab Accusamplerμ, VeruTech AB, Lund, Sweden) (catheterized mice). The mice were provided with aspen chip bedding (Tapvet, Kortteinen, Finland), shredded paper nesting material (Lilico, Horley, UK), bite bricks (Tapvey) and cardboard houses (Brogaarden, Gentofte, Denmark) for environmental enrichment. Feed (Altromin 1314; Altromin Gmbh & Co KG, Lage, Germany) and acidified tap water were provided ad libitum. A diurnal rhytm was maintained with a 12:12 hour light-dark cycle, starting with lights on at 6 a.m. Cage temperature was kept at 22°C, relative humidity at 55% and the air was exchanged approximately 75 times h^-1^.

The catheterized mice had in the preceding study been subjected to surgery with catheterization of the right common carotid artery and tunneling of the catheter subcutaneously to the nape of the neck. An analgesic regimen, consisting of 1 mg/kg body weight (BW) buprenorphine (Temgesic; Schering-Plough Europe, Brussels, Belgium) was given in nut paste (Nutella®, Ferrero, Pino Torinese, Italy) for voluntary ingestion prior to surgery and then once daily for two days post-surgery, as described previously [21,22]. To ensure adequate pre-emptive analgesia, the mice were injected with 0.1 mg buprenorphine/ kg BW before being brought out of anesthesia. After surgery, the mice were monitored every other hour for six hours and then twice daily for three days post-surgery.

In the ABS system, catheter patency was maintained through infusion of 65 μl of 25 IU/ml heparinized saline every 30 minutes. On the third day post-surgery, 25 μl blood samples were drawn automatically every third hour during the following 24 hours. The mice had been subjected to 15 minutes of video recording in a behavioral test, consisting of a combined open field, elevated plus maze and light-dark box [23].

Control mice were subjected only to 15 minutes of video recording in the same behavioral test, and had not been subjected to surgery or in life blood sampling. All animals were submitted for pathology immediately after termination of the preceding study, at which time all catheters were functional. For a full description of the preceding study, please refer to Teilmann *et al*. [7]

### Procedure

Upon termination of the preceding study, all mice were weighed and then placed in an induction chamber and anesthetized with 5% isoflurane in oxygen. When the hind paw withdrawal reflex was absent, the mice were exsanguinated by closed cardiocentesis. The mice were returned to the induction chamber, where the isoflurane concentration was maintained at 5% until cessation of respiration. The blood was collected in heparin-coated tubes (BD Microtainer; BD Inc., Franklin Lakes, USA) and centrifuged at 1000 g in a microcentrifuge (Labnet International Inc., Edison, NJ, USA) for 15 minutes to isolate plasma and a second time at 10,000 g for 10 minutes. The supernatant was transferred to clean microcentrifuge tubes and stored at—80°C until analysis for plasma cytokine concentrations.

The cytokines; interleukin-1 beta (IL-1β), interleukin-2 (IL-2), interleukin-6 (IL-6), interleukin-10 (IL-10), interleukin-17A (IL-17A), granulocyte macrophage colony-stimulating factor (GM-CSF), interferon gamma (IFN-γ) and tumor necrosis factor alpha (TNF-α) were quantified in duplicate using Bio-Plex Pro assays (Bio-Rad Laboratories, Copenhagen, Denmark) on a Luminex®100™ system (Ramcon, Birkerød, Denmark). A nine point standard dilution series was prepared and all assay reagents were prepared according to the manufacturer’s instructions. The lower limit of quantification (LLOQ) (calculated by the software) was defined as two standard deviations above the levels measured in the zero samples on the standard curve. The upper limit of quantification (ULOQ) was defined as the highest point on the standard curve with an intra-assay coefficient of variation (%CV) of less than 20% and with a recovery value between 70%-130%. The assay detection range was thus bounded by LLOQ and ULOQ. The assay principle has been described previously [24]

After euthanasia, the mice were subjected to necropsy and histopathologic sampling. All euthanasia, bleeding and necropsy procedures were performed between 9 am and noon. The heart, brain, spleen, liver, kidneys, adrenals and lungs, and surgical area at the ventral neck including the salivary glands were collected. The specimens were immersed in a 4% aqueous formaldehyde solution (Gurr® formaldehyde, VWR, Vienna, Austria). After approximately one month’s fixation, the surgical area including salivary glands was collected in one tissue block after the catheter was carefully removed. The area was dissected from side to side to include the midline surgery site intact. The heart was bisected longitudinally for evaluation of both ventricles and base of aorta. The brain was cross-sectioned, coronally, from dorsal to ventral using a razor blade at the levels of the olfactory bulb, cerebral cortex (neocortex), hippocampus and cerebellum, as described elsewhere [25]. The spleen was bisected longitudinally. Of the liver, two sections of the median lobe, including the gall bladder, were collected and the total number of microgranulomas was quantified in both cross sections of the median liver lobe for each mouse. Microgranulomas were scored only in the presence of focal granulocyte, sometimes mixed granulocyte-mononuclear cell, accumulations associated with hepatocyte necrosis, and were thus differentiated from extramedullary hematopoiesis (EMH), which is typically not associated with cellular degeneration or necrosis [26]. The left kidney was bisected longitudinally through the midline and the right kidney was transected near the midline. The lungs were inflated with formalin in situ before collection.

Trimmed tissue was embedded in paraffin [27]. A minimum of three sections for each organ, stained with hematoxylin and eosin, were examined using brightfield microscopy and scored in random order by two blinded pathologists. Furthermore, gram stains of liver sections from the mice with the highest numbers of microgranulomas were retrospectively evaluated (Mouse A-D).

### Statistics

Data were analyzed in SPSS Statistics 20 (IBM, Armonk, NY, USA) and analyzed for normality using Shapiro-Wilk’s tests. Normally distributed data sets were analyzed with an independent samples t-test for comparing the overall difference between groups. Levene’s test of equality of error variances was conducted to test that the variances were equal across groups. Statistics are presented as a t-value, t(df), where df are the degrees of freedom. The data points of the cytokines IL-6 and IFN-γ did not conform to a Gaussian distribution and were thus subjected to a Mann-Whitney U test. Statistics are presented as a U value, as well as the asymptotic significance (2-tailed) *p*-value. For weight data p-values < 0.05 were considered significant. To account for multiple comparisons, a narrower p-value of < 0.01 was considered significant for cytokine concentrations.

## Results

### Cytokines

Catheterized mice did not express significantly different concentrations of the cytokines IL-1β (*t*(7.263) = -0.162, *p* = 0.876); IL-2 (*t*(6.000) = 1.549, *p* = 0.172); IL-10 (*t*(6.617) = -0.510, *p* = 0.626); IL-17A (*t*(10) = -1.550, *p* = 0.152); GM-CSF (*t*(13) = -1.294, *p* = 0.218); TNF-α (*t*(7.229) = -0.303, *p* = 0.771) and IFN-γ (*U* = 20, *p* = 0.319) compared to control mice (Table 1). The concentrations of IL-6 were significantly elevated in catheterized mice compared to control mice (*U* = 8, *p* = 0.006). Raw data are given in S1 Table.

**Table 1:** Cytokine levels in control mice and catheterized mice.

	Control mice		Catheterized mice	
Cytokines	Mean	SD	Mean	SD
IL-1β	0.164	0.033	0.170	0.097
IL-2	0.010	< 0.001	0.007	0.005
IL-10	0.071	0.012	0.081	0.051
IL-17A	0.073	0.031	0.110	0.052
GM-CSF	0.100	0.029	0.122	0.039
TNF-α	0.414	0.058	0.434	0.171
IFN-γ	0.015	0.005	0.024	0.018
**IL-6**	**0.010**	**< 0.001**	**0.034**	**0.039**

Mean cytokine concentrations (pg/ml) and standard deviations (SD) are given for each cytokine. The concentrations of only one cytokine, IL-6, was significantly different between groups, where catheterized mice (N = 7) had elevated levels of IL-6 compared to control mice (N = 8, Mann-Whitney, p = 0.006).

### Body weights

BWs of control mice, 39.2 ± 3.6 (mean ± SD), and catheterized mice, 35.2 ± 3.4, did not differ significantly (t(12) = 2.109, *p* = 0.057) at the time of euthanasia, although catheterized mice on average weighed less. Likewise, the BW change from the start of the preceding study [7] until euthanasia did not differ between groups (*U* = 14.000, *p* = 0.103). Raw data are given in S2 Table.

### Pathology

In Table 2, histopathology changes in the examined tissues are summarized. No gross pathological changes were identified in any mice.

**Table 2:** Histopathological scorings.

Group	Catheter							Control							
Mouse identification	A	B	C	D	E	F	G	H	I	J	K	L	M	N	O
**Surgical area and salivary glands**															
Adipose and connective tissue															
*Inflammation*	X	X	X	X	X	X	X				X				
*Fibroblast proliferation*	X	X	X	X	X	X	X								
*Arteritis with thrombosis*				X	X										
*Angiogenesis*				X											
*Necrosis*						X									
*Edema*						X									
*Necrosis and regeneration of skeletal muscle*		X		X		X	X								
Submandibulary gland															
*Inflammation*		X			X	X	X				X				
*Degenerative changes*	X	X			X		X								
*Necrosis*					X	X	X								
Sublingual gland															
*Inflammation*	X	X				X									
*Degenerative changes*	X					X									
*Necrosis*															
Parotid gland															
*Inflammation*		X			X		X								
*Degenerative changes*	X				X	X	X								
*Necrosis*	X	X			X		X								
**Heart**															
LVFW															
*Degeneration of cardiomyocytes*					X		X								
IVS															
*Degeneration of cardiomyocytes*					X		X								
*Inflammation*							X								
*Fibroblast proliferation*							X								
RVFW															
*Degeneration of cardiomyocytes*					X		X								
**Kidneys**															
*Vacuolization of tubular epithelium*	X	X	X	X			X		X						
*Tubular regeneration*	X	X	X	X			X								
*Tubular ectasia*		X	X	X			X								
*Cystic degeneration*		X		X			X								
*Perivasculitis*							X								
*Glomerular necrosis*							X								
*Inflammation*	X	X					X		X		X		X		
**Liver**															
*Microgranulomas*	37	6	5	6	1				1						
*Single-cell necrosis of hepatocytes*	X	X	X			X									
*Diminished glycogen accumulation*	X	X		X		X									

The table shows the pathological changes, observed in individual catheterized (N = 7) and control (N = 8) mice. LVFW = left ventricular free wall, IVS = interventricular septum, RVFW = right ventricular free wall. The number of microgranulomas, counted in two cross-sections of the median liver lobe for each mouse, is also given. No lesions could be identified in the spleen, adrenals, brains or lungs of any mice, and these organs are therefore not shown in the table.

**Fig 1:**
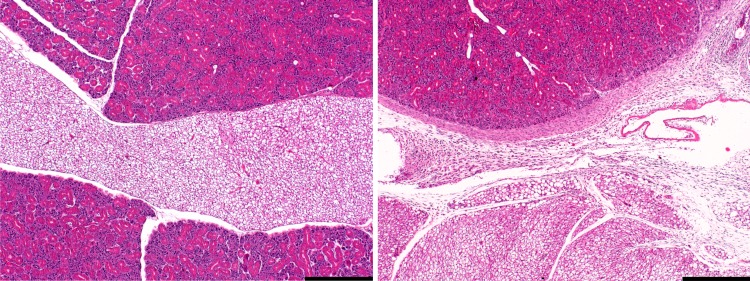
The surgical area of the ventral neck in one control mouse and one catheterized mouse. Normal histology (left image) shows a midline area of connective and adipose tissue and the bilateral submandibulary salivary glands. In the right image, the surgical area of one catheterized mouse is affected by increased clear space (edema) and infiltration of inflammatory cells. Fibroblast proliferations suggest regenerative processes. Hematoxylin and eosin. Bars = 500 μm.

**Fig 2:**
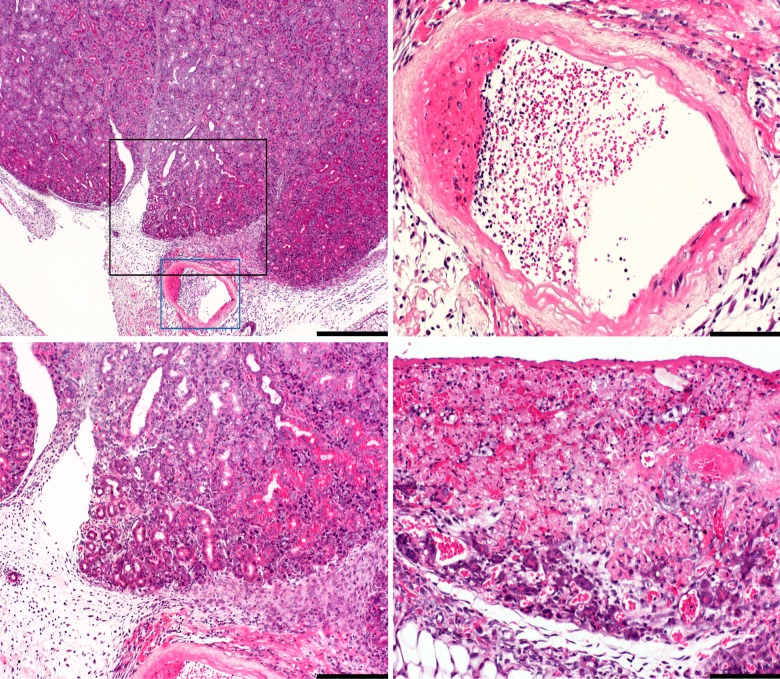
Surgical area at the ventral midline of catheterized mice. In the top left image, the submandibulary salivary gland is multifocally degenerated to necrotic with loss of zymogen granules in tubules and acini of this male mouse. There is increased clear space (edema) in the connective and adipose tissue with infiltration of inflammatory cells (inflammation) and proliferation of fibroblasts (regeneration). The wall of a major artery is infiltrated with inflammatory cells (arteritis) with thrombus formation, protruding to the arterial lumen. The areas highlighted by the black and blue boxes are shown in higher magnification in the bottom left and top right images, respectively. Another catheterized mouse (bottom right image) was found with similar lesions; the submandibulary salivary gland, adjacent to a major artery, was found compressed and necrotic with hemorrhage and neovascularization (angiogenesis) in the necrotic area. Hematoxylin and eosin. Bars = 500 μm in top left image, 200 μm in bottom left image and 100 μm in both right images.

Surgical sites in all catheterized mice had mild to moderate granulocytic and lymphocytic infiltration of adjacent connective tissue, occasionally also involving the surrounding skeletal muscle. These changes were mostly evident in the midline, but extended laterally in few mice, with fibroblast and myofibroblast proliferation consistent with healing (Fig 1). In two catheterized mice mild arteritis and thrombi were identified near the catheter sites with angiogenesis of granulation tissue in this area of one mouse (Fig 2).

Submandibular salivary glands in five catheterized mice had degenerative to necrotic changes with inflammation in four of these mice. These changes were predominantly unilateral, near the catheter tunnel. The ipsilateral right sublingual salivary gland in five catheterized mice had chronic and active inflammation, with degenerative acinar changes in two of these mice. The parotid gland in five catheterized mice had degenerative to necrotic acinar changes, and was unilaterally infiltrated with chronic and active inflammation in three catheterized mice. One control mouse had mild, multifocal, chronic lymphoplasmacytic inflammation involving the subcutis and adjacent submandibular gland with no source or cause identified.

The hearts of two catheterized mice had multifocal, mild cardiomyocyte degeneration, loss and replacement (fibrosis) in the left ventricular free wall (LVFW), the interventricular septum (IVS) and the right ventricular free wall (RVFW) (cardiomyopathy). One of these mice had mild associated granulocytic and lymphocytic inflammation. Heart lesions were not identified in the control mice.

**Fig 3:**
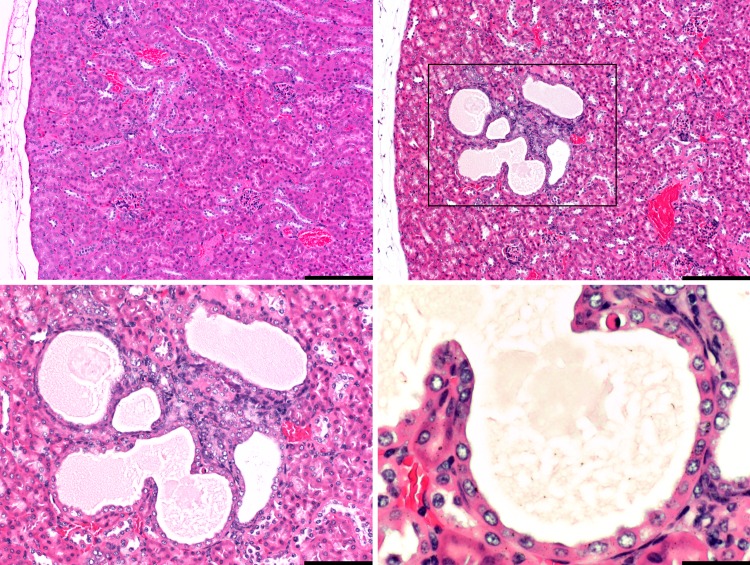
Kidney from one control mouse and one catheterized mouse. The top left image shows normal kidney histology in one control mouse. In the top right image, focal tubular degeneration with cyst formation is seen, shown in higher magnifications in the bottom left and right images, as indicated in the black and blue boxes, respectively. The epithelium is plump and hyperbasophilic, suggesting regeneration. Note the apoptotic epithelial cell in one dilated tubule (bottom right image). Bars = 200 μm in the top images, 100 μm in the bottom left image and 33 μm in bottom right image.

Kidneys of five catheterized mice had multifocal, mild cortical degenerative and regenerative changes and mild chronic inflammation with cystic tubule dilatation up to 1 mm in diameter (cystic degeneration) in three of these mice (Fig 3). One catheterized mouse had inflammation around arcuate arteries and veins (perivasculitis) with necrosis of two adjacent glomeruli. One catheterized mouse had a fibrinocellular arterial thrombus in adjacent perirenal muscle. In three control mice, focal, lymphocytic inflammation was noted at the renal pelvis.

**Fig 4:**
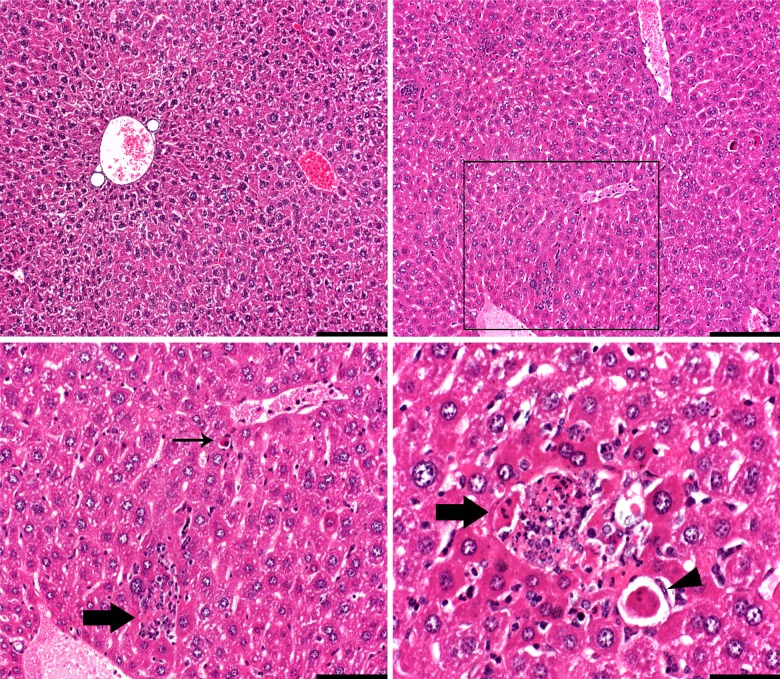
Liver from one control mouse and one catheterized mouse. The top left image shows normal liver histology in one control mouse. In the top right image, multifocal granulocyte accumulations in association with hepatocyte necrosis (microgranulomas) are seen, shown in higher magnifications in the bottom images (highlighted by thick arrows). The area highlighted in the black box is shown in higher magnification in the bottom left image. Note the apoptotic hepatocyte (thin arrow). The bottom right image shows another microgranuloma in the same mouse (not shown in the top right image). Note the single cell necrosis in the bottom right corner (arrow head). Bars = 200 μm in the top images, 100 μm in the bottom left image, and 50 μm in the bottom right image.

The livers of four catheterized mice had multifocal, randomly distributed, focal lymphohistiocytic inflammatory infiltrates, sometimes with intrahistiocytic pigment or degenerate cells, in association to hepatocyte necrosis (microgranulomas), where up to 37 microgranulomas were identified in sections from one of these mice (Fig 4). One catheterized and one control mouse had one microgranuloma in the studied sections. Four catheterized mice had prominent single cell necrosis, often in close proximity to microgranulomas. Gram stains were negative for microorganisms.

No changes could be identified in the spleens, adrenals, brains or lungs of the mice in either group.

## Discussion

We have previously shown that ABS through a vascular catheter may be applied in rodents with minimal effects on multiple physiological parameters and behaviors [5,7,14]. However, a vascular catheter provides direct access to the circulation and should constantly be regarded as a potential source of infection. Fonseca *et al*. demonstrated kidney infection and inflammation in catheterized rats seven days post-surgery [12], which impacts the utility of chronically catheterized rodents in studies where kidney function is important for the study outcomes, and contributes to our concern that other tissues also may be impacted by chronic catheterization.

Fonseca *et al*. studied the isolated effect of the implantation and presence of a vascular catheter, without interfering with the catheters until the end of study. Many automated systems (ABS) are sterilized before connecting a new animal to the machine, but cannot be kept completely sterile during the study period, which may lead to bacterial colonization of tubing over time. Thus, regular flushing and blood sampling through the catheter, as performed automatically in an ABS system, may enhance the risk of introducing bacteria, which may embolize to various tissues. Assessment of the inflammatory and tissue level effects of chronic catheterization with automated blood sampling is important in order to optimize the use of ABS in rodent research, drug discovery and pre-clinical testing.

The pro-inflammatory cytokines IL-6, IL-1 and TNF-α act on the central nervous system to induce a complex set of behaviors and physiological responses, collectively termed the *illness responses*, which promote immune defense and tissue repair through reducing energy expenditure and increasing body temperature [28]. These responses may be clinically subtle and easily overlooked in the laboratory mouse; fever, increased sleep and decreased activity, decreased food–and water intake and reduced social interaction [29]. Therefore, measuring pro-inflammatory cytokines can offer valuable information regarding an animal’s health and well-being.

In the present study, only the plasma concentration of IL-6 differed between groups, where catheterized mice had higher levels than control mice. Surgery is a major inducer of IL-6 [16,30], where IL-6 is detectable in the circulation within 30–60 minutes after surgery and may persist for up to ten days, depending on the severity of the tissue damage. IL-6 is also secreted at the site of endothelial damage and is involved in thrombogenic activity [17,31]. Furthermore, IL-6 is secreted in response to other stimuli such as psychological stressors (open field, immobilization) [18,19]. In the present study, increased IL-6 levels in catheterized mice likely reflected a physiologic response to surgery, but local irritation of the endothelium by the catheter, stress responses to the surgery, post-surgical recovery, the ABS system, and the final behavioral test may have contributed as well.

While IL-1β and TNF-α are also secreted in response to tissue injury, these cytokines are released immediately as part of the acute phase response, which subsides within 24–48 hours, and are thus only briefly present in the circulation [16], unless other stimuli, such as chronic-active inflammation or post-surgical pain, maintains elevated cytokine levels in circulation [15]. The concentrations of IL-1β and TNF-α were not significantly elevated. Likely, these cytokines increased following surgery, but normalized before the time of necropsy in the absence of ongoing acute inflammatory stimuli.

IL-10, IL-17A, IL-2, IFN-γ and GM-CSF reflect different stages of immune activation, usually related to the presence of pathogens [32–36]. They were included to assess the degree of post-operative complications such as infection, or other causes of excessive or inappropriate inflammatory responses, which were not identified. Therefore, based on cytokine quantification, it is assumed that the peak of the *illness response* following surgery had passed at the time of necropsy, four days post-surgery, and that the catheterized mice had recovered with no measurable signs of systemic inflammation.

Histopathology confirmed tissue damage remote from the surgical site. At the surgical sites, modest inflammation and fibroplasia were consistent with aseptic surgery and the four-day post-operative period.

Salivary gland changes at the side of catheter tunneling, sometimes with tissue necrosis, were generally modest but exceeded expected changes and suggest that catheterization may affect salivary gland vascular supply or induce acinar necrosis from local compression by the catheter. Salivation plays an important role in food consumption and digestion in rodents [37], and have roles in host defense [38]. Thus, salivary gland impairment may potentially impact food intake, body weight and some innate defense mechanisms. Food consumption was found to decline post-surgically in the preceding study but to reach pre-operative levels by three days post-surgery. Likewise, body weights of catheterized mice were found to decrease post-surgically in the preceding study [7], but were not found to be significantly different from those of the control mice in the present study. We attributed the transitional decline in food consumption and body weights of catheterized mice to the surgery and possibly also the administration of buprenorphine, as reduced food consumption is often seen in the post-surgical recovery period to other surgical procedures [39–41] and because buprenorphine is known to decrease food consumption [42,43]. Supporting these theories, the food intake and body weights normalized in step with surgical recovery and termination of analgesic administration. However, the chronic effects of salivary gland necrosis, even unilateral, on the rodent model have to our knowledge not been examined. These effects should be considered in studies where salivary function, food consumption or digestion are relevant.

Cardiac changes related to catheterization may have clinical and experimental effects. However, myocardial degeneration may occur spontaneously in older mice [44] and further investigation is necessary to appropriately elucidate the effects of carotid catheterization on cardiac function in mice. A follow up study is currently in progress.

Kidney changes in five catheterized mice and the thrombus in one of these suggest ischemia related to disseminating microthrombi as likely causes or contributors to these findings. Both catheterized mice and controls had mild chronic (non-suppurative) inflammatory changes, which are common background findings in laboratory rodents [45]. Conversely, inflammation associated with the degenerative changes in catheterized mice was interpreted as relevant to tissue injury. One control mouse was found to have mild, multifocal, randomly distributed vacuolization of tubular epithelium consistent with lipid vacuolation, which are often seen in mice of heavy breeds such as NMRI [46], and was considered non-specific and unrelated to the study.

Liver microgranulomas were increased in catheterized compared to non-catheterized mice. Few microgranulomas, consisting of small lymphocytic and histiocytic infiltrates, are common incidental findings in mice in all age groups [47]. Increased presence of microgranulomas, however, has been suggested to be caused by disturbance of microvascular dynamics [48] or by chronic infection with *Helicobacter sp*. or murine norovira [26]. Often, these aggregates are associated with increased single hepatocyte necrosis [46] compared to usually inconspicuous apoptosis related to controlled removal of senescent hepatocytes [49]. Although *Helicobacter sp*. are present in mouse facilities in our department, high numbers of microgranulomas and increased single hepatocyte necrosis were identified in four catheterized mice only, and contrasted clearly with the control mice.

In conclusion, chronically catheterized ABS mouse models can mitigate stress and facilitate multiple blood collections relevant to diverse research areas but, as demonstrated in the present study, potential confounding effects of visceral damage should be considered and assessed.

## Supporting Information

S1 Table Raw data. **Plasma cytokine concentrations.** The table provides plasma concentrations (pg/ml) of the cytokines; interleukin-1 beta (IL-1β), interleukin-2 (IL-2), interleukin-6 (IL-6), interleukin-10 (IL-10), interleukin-17A (IL-17A), granulocyte macrophage colony-stimulating factor (GM-CSF), interferon gamma (IFN-γ) and tumor necrosis factor alpha (TNF-α) of catheterized (Cath, N = 7) and control mice (N = 8). Samples below the detection limit are indicated by < 0.01.
(DOCX)

S2 Table Raw data. **Body weights.** The table shows the body weights (gram) of catheterized (Cath, N = 7) mice and control mice (N = 8) at the beginning of the preceding study (BW_pre_) and at the time of euthanasia (present study, BW_eut_).
(DOCX)
