# Supplementary material for: Carotid Catheterization and Automated Blood Sampling Induce Systemic IL-6 Secretion and Local Tissue Damage and Inflammation in the Heart, Kidneys, Liver and Salivary Glands in NMRI Mice
Source: PLoS One. 2016 Nov 10;11(11):e0166353. doi: 10.1371/journal.pone.0166353 (PMC5104411; doi:10.1371/journal.pone.0166353)
Supplement: S1 Table — Plasma cytokine concentrations. The table provides plasma concentrations (pg/ml) of the cytokines; interleukin-1 beta (IL-1β), interleukin-2 (IL-2), interleukin-6 (IL-6), interleukin-10 (IL-10), interleukin-17A (IL-17A), granulocyte macrophage colony-stimulating factor (GM-CSF), interferon gamma (IFN-γ) and tumor necrosis factor alpha (TNF-α) of catheterized (Cath, N = 7) and control mice (N = 8). Samples below the detection limit are indicated by < 0.01. (DOCX) [file pone.0166353.s001.docx]

S1 Table. Raw data. Plasma cytokine concentrations.

| **Group** | **Mouse ID** | **IL-1β** | **IL-2** | **IL-6** | **IL-10** | **IL-17A** | **GM-CSF** | **IFN-γ** | **TNF-α** |
| --- | --- | --- | --- | --- | --- | --- | --- | --- | --- |
| **Cath** | A | 0.21 | 0.01 | 0.02 | 0.12 | 0.11 | 0.10 | 0.06 | 0.59 |
|  | B | 0.29 | 0.01 | 0.02 | 0.15 | 0.17 | 0.16 | 0.03 | 0.64 |
|  | C | 0.20 | 0.01 | 0.02 | 0.09 | 0.10 | 0.13 | 0.02 | 0.47 |
|  | D | 0.27 | 0.01 | 0.04 | 0.12 | 0.14 | 0.19 | 0.03 | 0.54 |
|  | E | 0.10 | 0,01 | 0.12 | 0.04 | 0.03 | 0.10 | 0.01 | 0.33 |
|  | F | 0.08 | <0.01 | 0.01 | 0.03 | <0.01 | 0.09 | 0.01 | 0.29 |
|  | G | 0.04 | <0.01 | 0.01 | 0.02 | <0.01 | 0.09 | 0.01 | 0.18 |
| **Control** | H | 0.15 | 0.01 | 0.01 | 0.06 | <0.01 | 0.15 | 0.01 | 0.38 |
|  | I | 0.16 | 0.01 | 0.01 | 0.06 | 0.05 | 0.10 | 0.01 | 0.44 |
|  | J | 0.12 | 0.01 | 0.01 | 0.07 | 0.05 | 0.12 | 0.01 | 0.35 |
|  | K | 0.20 | 0.01 | 0.01 | 0.09 | 0.11 | 0.12 | 0.02 | 0.50 |
|  | L | 0.20 | 0.01 | 0.01 | 0.09 | 0.10 | 0.09 | 0.02 | 0.39 |
|  | M | 0.20 | 0.01 | 0.01 | 0.07 | 0.10 | 0.07 | 0.02 | 0.48 |
|  | N | 0.12 | 0.01 | 0.01 | 0.06 | 0.03 | 0.09 | 0.01 | 0.34 |
|  | O | 0.16 | 0.01 | 0.01 | 0.07 | 0.07 | 0.06 | 0.02 | 0.43 |

The table provides plasma concentrations (pg/ml) of the cytokines; interleukin-1 beta (IL-1β), interleukin-2 (IL-2), interleukin-6 (IL-6), interleukin-10 (IL-10), interleukin-17A (IL-17A), granulocyte macrophage colony-stimulating factor (GM-CSF), interferon gamma (IFN-γ) and tumor necrosis factor alpha (TNF-α) of catheterized (Cath, N = 7) and control mice (N = 8). Samples below the detection limit are indicated by < 0.01.
