# Supplementary material for: Carotid Catheterization and Automated Blood Sampling Induce Systemic IL-6 Secretion and Local Tissue Damage and Inflammation in the Heart, Kidneys, Liver and Salivary Glands in NMRI Mice
Source: PLoS One. 2016 Nov 10;11(11):e0166353. doi: 10.1371/journal.pone.0166353 (PMC5104411; doi:10.1371/journal.pone.0166353)
Supplement: S2 Table — Body weights. The table shows the body weights (gram) of catheterized (Cath, N = 7) mice and control mice (N = 8) at the beginning of the preceding study (BWpre) and at the time of euthanasia (present study, BWeut). (DOCX) [file pone.0166353.s002.docx]

**S2 Table. Raw data. Body weights.**

| **Group** | **Mouse ID** | **BW_pre_** | **BW_eut_** |
| --- | --- | --- | --- |
| **Cath** | A | 42.0 | 39.1 |
|  | B | 39.0 | 36.6 |
|  | C | 37.5 | 29.8 |
|  | D | 38.5 | 34.6 |
|  | E | 36.5 | 33.1 |
|  | F | 38.0 | 38.8 |
|  | G | 37.0 | 38.1 |
| **Control** | H | 45.5 | 45.5 |
|  | I | 34.5 | 35.5 |
|  | J | 36.0 | 36.0 |
|  | K | 40.0 | 40.5 |
|  | L | 40.5 | 41.5 |
|  | M | 37.5 | 38.0 |
|  | N | 36.0 | 35.5 |
|  | O | 41.5 | 41.5 |

The table shows the body weights (grams) of catheterized (Cath, N = 7) mice and control mice (N = 8) at the beginning of the preceding study (BW_pre_) and at the time of euthanasia (present study, BW_eut_).
